# Leaving no one behind: health equity as a catalyst for the sustainable development goals

**DOI:** 10.1093/eurpub/ckaa033

**Published:** 2020-05-11

**Authors:** Tatjana Buzeti, Joana Madureira Lima, Lin Yang, Chris Brown

**Affiliations:** WHO European Office for Investment for Health and Development, Venice, Italy

## Abstract

2019 has been a milestone year for catalyzing changes to improve health equity in the WHO European Region through concomitant progress in the sustainable development goal (SDG) targets. The WHO European Health Equity Status Report Initiative (HESRi) has captured and analyzed the relationships between inequities in health and the conditions that are essential for all to be able to live healthy and prosperous lives. The five essential conditions map directly onto a number of SDG targets, with a much broader span than SDG3 on health. They are: (i) Universal access to good-quality, affordable health services; (ii) Basic income security and social protection; (iii) Safe and decent living conditions; (iv) Inclusive social and human capital building opportunities; and (v) Decent and non-discriminatory employment and working conditions. There is certainly room for improvement in the way ahead, particularly in the availability of fine-grained and disaggregated data, and in the quality of monitoring and analysis of policy options that this would allow. However, the work of the HESRi shows that by harnessing such data it is possible to show what actions policymakers can take in the present to ensure that no one is left behind. This equity framing allows to measure whether the progress on SDGs benefits all, including those who need them most.

## Leaving no one behind commitment

The commitment to ‘leave no one behind’ is a core principle of the 2030 Agenda for Sustainable Development and the sustainable development goals (SDGs). ‘Leaving no one behind’ puts equity at the centre of national and international policy agendas, representing a critical improvement over the previous set of Millennium Development Goals^1^ which were largely silent on the issue of social disparities.

Although the commitment to leave no one behind is seldom disputed in principle, converting principle into practice is a complex task. To achieve a widespread paradigm shift towards development that leaves no one behind, transformation of deeply rooted systems is necessary. These systems are often based on unequal distributions of financial and decision-making power, and include economic, social and political systems, governance structures and business models. Identifying an entry point to tackle these interlinked systems of power can be critical for focussing action and catalyzing progress, alongside availability of a monitoring instrument and roadmap for converting equity principles into measurable targets and policy actions.

The rest of this article discusses exactly such a policy instrument, one which takes health equity as its entry point—the Health Equity Status Report Initiative (HESRi). Given the nature of health as a basic building block for life chances, a roadmap for leaving no one behind in health provides the foundations and signposts the pathways to leaving no one behind in life and in achieving the SDGs.

## How do the SDGs contribute towards improving health equity?

2019 has been a milestone year for catalysing changes to improve health equity in the WHO European Region through concomitant progress in the SDG targets. The WHO European HESRi has captured and analyzed the relationships between inequities in health and the conditions that are essential for all to be able to live healthy and prosperous lives. The five essential conditions map directly onto a number of SDG targets, with a much broader span than SDG3 on health:

- Universal access to good-quality, affordable health services (SDG3)
- Basic income security and social protection (SDG1 and 10)
- Safe and decent living conditions (SDG2, 6, 7 and 11)
- Inclusive social and human capital building opportunities (SDG4, 5 and 16)
- Decent and non-discriminatory employment and working conditions (SDG5 and 8)

The European Health Equity Status Report (HESR),^2^ the core document of the HESRi, uses decomposition analysis to quantify the relative magnitude of each condition’s contribution to health inequities, taking into account the contribution of each other condition. Inequities are measured in three outcome indicators—self-reported health, mental health and life satisfaction. The analysis shows that the contribution of each of the five conditions to health inequities is statistically significant. The relative size of their contributions is largely consistent across the indicators—e.g. systematic differences between socioeconomic groups in income security and in living conditions are the largest contributors to inequities in self-reported health, mental health and life satisfaction within countries of the WHO European Region. The relative contributions of living conditions to inequities in self-reported health, mental health and life satisfaction between the most and least disadvantaged are 29%, 30% and 21%, respectively. The relative contributions of health services to inequities in self-reported health, mental health and life satisfaction between the most and least disadvantaged are 10%, 11% and 11%, respectively.

These findings send a strong message: solutions to reduce health inequities should be:

- Cross-sectoral—Ministries of Health working alone will not be able to deliver on equity. Progress must rely on collaboration among Ministries across government, with non-state actors and civil society;
- Comprehensive—no single policy option in isolation, even with evidence of effectiveness, will suffice;
- Coherent across the policy portfolio—the aims and implementation of a policy in one sector of society, e.g. economic growth, cannot undermine the equity goals of another policy, e.g. labour protection.

Given that all five essential conditions correspond with SDG targets, the HESRi presents itself as a tool for answering ‘How do the SDGs contribute towards improving health equity?’ and explores, ‘How does a health equity frame contribute to improving progress on the SDGs?’ This later question is crucial in leaving no one behind because the SDGs do not consistently incorporate measures of equity when measuring their progress. Consequently, there is a risk that the benefits reaped by advancing the SDG agenda are not shared by those who need it most. Elements of the HESRi, such as the Health Equity Policy Tool,^3^ make explicit link between SDG targets, health equity, and measures of equity in the essential conditions needed to make progress in both and are thus valuable in measuring progress for those left behind.

## A health equity perspective on the monitoring and reporting of the SDGs

The HESRi harnesses micro data at the level of the individual to assess the relative contributions of the five essential conditions to health inequities, and also to produce the disaggregated data necessary for monitoring of status and trends in health inequities. There is still, considerable variability from one Member State to another in the availability of disaggregated data for such monitoring—be it disaggregated by income, education, geographic area or other marker. This means it is not straightforward to monitor the evolution of health gaps consistently across *all* countries of the WHO European Region, which is necessary for the reporting of SDG indicators.

In monitoring SDG3—Ensure healthy lives and promote well-being for all at all ages—some SDG3 indicators have an equity dimension. Indicator 3.8.1 implicitly recognizes the need to collect data on the progress of the most disadvantaged: ‘Coverage of essential health services (defined as the average coverage of essential services based on tracer interventions that include reproductive, maternal, new-born and child health, infectious diseases, non-communicable diseases and service capacity and access, among the general and the most disadvantaged population).’ Similarly, indicator 3.8.2 ‘Proportion of population with large household expenditures on health as a share of total household expenditure or income’ has a built-in equity dimension as it acknowledges that those on lower incomes are more likely to be impoverished as a consequence of paying for health-care out of pocket (OOP).

Nonetheless, this equity perspective is not present across all of the indicators deployed to measure progress. The danger with equity-blind progress monitoring is that it is technically possible to achieve progress that is driven by those who might have already been doing well. In other words, progress that is not evenly distributed across all in society. As an example, target **3.9** is ‘By 2030, substantially reduce the number of deaths and illnesses from hazardous chemicals and air, water and soil pollution and contamination.’ Its indicator **3.9.1** ‘Mortality rate attributed to household and ambient air pollution’ poses a challenge to monitoring gaps in inequities in mortality rates. An equity sensitive indicator would require data collection with a level of disaggregation that recognizes that those living in settings with fewer resources may be more exposed to pollution. They are also less likely to move to healthier settings as a result, and thus at higher risk of developing fatal health conditions from ambient air pollution.

Mainstreaming health equity into the monitoring of progress ensures that priority is given to accelerating the rate of progress where it is most needed. This means that the relative health gains would be greater for those most left behind, which in turn benefits the whole of society.

## Accelerating progress towards equity within the SDGs

The HESRi provides tools for policymakers, practitioners and advocates alike. It goes beyond the monitoring of health inequity across the Region by offering policy options for narrowing health inequities, through smarter investment and more effective coverage and uptake of policies promoting the conditions necessary for health equity. The HESR assesses the relationships between health equity and eight macro-economic policies within short-term time frames, up to 4 years.

Six of the eight policies analyzed show a significant statistical association with reductions in health inequities. This indicates their potential as policy levers to close the health gaps. The magnitude of the association with health inequity of each of these policies is different. The six policies showing statistically significant potential to reduce inequities in limiting illness among adults, in the short term, are: increases public expenditure on housing and community amenities; increases in expenditure on labour market policies; increases in social protection expenditure; reductions in OOP payments for health; reductions in unemployment; and reductions in income inequality. Increases in per-capita income show no association with reductions in health inequities, while increases in public expenditure on health show a positive association, however, unlike reductions in OOP, this association is not statistically significant.

The analysis shows that the gaps in health between socioeconomic groups can be reduced, even within political mandates of 4 years. Policymakers can feel confident that with the right investments and interventions it is possible to reduce inequities in health, even in the short term.

This policy analysis, along with the decomposition analysis introduced earlier, is a catalyst for action to accelerate progress towards not only narrowing health inequities but towards achieving a broad range of the SDGs. The rest of this section provides examples outlining some of the policy options put forward by the HESR that have the potential to reduce inequities in the targets of SDG1, 3, 5 and 10.

### SDG1—end poverty in all its forms everywhere, and SDG10—reduce inequality within and among countries

The HESR decomposition analysis shows that out of the total contribution of the five conditions to inequity in self-reported health between the most and least affluent 20% of adults within countries of the WHO European Region, on average 35% is due to systematic differences in risk and exposure to income insecurity and the lack or inadequacy of social protection. The HESR policy analysis corroborates with these findings, showing that increases in social protection expenditure are associated with reductions in health inequity. However, in the majority of WHO European Region countries, trends in social protection show that spending has either not significantly changed or even decreased over recent years.

These analyses can be mapped directly to SDG target 1.3 (‘Implement nationally appropriate social protection systems and measures for all, including [minimum protection] floors, and by 2030 achieve substantial coverage of the poor and the vulnerable’). The analyses show that aside from being a target for SDG1 in its own right, this target has a health equity-promoting dimension, and progress towards both SDG1 and reducing health inequity through investment in social protection. In particular, non-stigmatizing social protection policies have positive effects on reducing health inequities related to income insecurity and poverty. Robust, multilevel, inclusive income security systems—with a base tier of unconditional, non means-tested social security supplemented by state-supported contributory schemes—are needed to effectively reduce health inequities. These schemes include well-designed parental leave policies, statutory pensions, social protection for early years and families and unemployment benefits.

### SDG 3—ensure healthy lives and promote well-being for all at all ages

Out of the total contribution of the five conditions to inequity in self-reported health between the most and least affluent 20% of adults within countries of the WHO European Region, on average 10% is due to systematic differences in the quality, availability and affordability of health services. In the majority of countries across the WHO European Region, inequities in unmet need for health care either remained unchanged or increased between 2008 and 2017. The mean difference in rates of unmet need for health care between those with most and fewest years of education increased from 2.6 to 2.7 percentage points between 2008 and 2017 in countries across the Region.

The report finds a social gradient in catastrophic health spending, with those in the poorest quintile being more likely to experience catastrophic health spending compared with those in higher quintiles. For the 24 countries for which there was data, on average four more people out of every 100 in the poorest consumption quintile experience catastrophic health spending compared with those in the highest consumption quintile. In many countries the difference between the lowest quintile and the next income quintile is large, showing it is the poorest in society who are experiencing the worst effects of catastrophic health spending.

The HESR puts forward policy options that can be deployed to both achieve SDG3 and reduce these inequities. For example, reductions in OOP payments for health, which the policy analysis shows to have a significant statistical association with reductions in health inequity, also supports target 3.8 (‘Achieve universal health coverage, including financial risk protection, access to quality essential health-care services and access to safe, effective, quality and affordable essential medicines and vaccines for all’) through progress in indicator 3.8.2 (‘Proportion of population with large household expenditures on health as a share of total household expenditure or income’).

### SDG 5—achieve gender equality and empower all women and girls

Gender inequalities intersect with other forms of discrimination, contributing to inequities in income, living conditions, social and human capital, work and employment. Addressing these inequities is a prerequisite for achieving universal health coverage.

Cultural norms and practices such as the roles and rights of women and men and of girls and boys can magnify the health equity effects of the above determinants. Gendered roles and stereotypes begin in early childhood, when children start socializing and continue throughout life resulting in gender inequalities that limit women’s power and access to resources while contributing to the gender- unequal division of paid and unpaid work. They also contribute to the excess exposure to health risks among men, such as substance abuse, suicide and violence and to the biases in the response from the health sector.

Men’s and women’s behaviours, exposure to risk and health-seeking patterns are influenced by many factors, including the place they live and their employment situation, education, cultural context and social networks. Growing evidence suggests that factors affecting notions of masculinity and femininity and the way gender roles are defined in societies have a massive effect on the health of men and women in the European Region.

Improving the health and well-being of men and that of women are complementary objectives that are best addressed within a gender equality framework. Engaging men and facilitating their participation in paid and unpaid care, prevention of violence against women and sharing responsibility for reproductive health are key interventions needed to achieve global goals on gender equality and to accelerate progress in achieving health goals.^4^^,^^5^

Some progress has been made in recent years to end discrimination against women and girls in laws, policies and practice,^6^ but currently there is no comprehensive overview of data on legal frameworks in place to promote, enforce and monitor equality and non-discrimination on the basis of sex.

## Ways forward

The challenges of accurate data, disaggregated by age, sex, income, disability, geographic location, education and other social characteristics are not only evident in terms of SDG 3, but particularly relevant to linkages with SDGs 1, 4, 5, 10, 16 and 17 from the perspective of addressing the highest needs of vulnerable populations and leaving no-one behind.

Those who are left behind are exposed to multiple risks across different SDGs. To drive progress towards leaving no one behind from a health equity framing, data and evidence are required for measuring health and well-being status, relationships with the underlying conditions and the coverage and investment in policies. This means taking a pathways approach in SDG monitoring. The HESRi online interactive data platform is a step into this direction. In line with the ethos of SDG target 17.18, it is openly accessible in the public domain (available at https://whoeurope.shinyapps.io/health_equity_dataset/).^7^

## Disclaimer

The authors alone are responsible for the views expressed in this article, and they do not necessarily represent the views, decisions or policies of the institutions with which they are affiliated.

*Conflicts of interest*: The author is a staff member of the WHO Regional Office for Europe. The author alone is responsible for the views expressed in this publication and they do not necessarily represent the decisions or the stated policy of the World Health Organization.
